# SOS to the Soccer World. Each Time the Preseason Games Are Less Friendly

**DOI:** 10.3389/fspor.2020.559539

**Published:** 2020-12-18

**Authors:** Julio Calleja-Gonzalez, Carlos Lalín, Francesc Cos, Diego Marques-Jimenez, Pedro E. Alcaraz, Antonio José Gómez-Díaz, Tomás T. Freitas, Juan Mielgo Ayuso, Irineu Loturco, Xavi Peirau, Ignacio Refoyo, Nicolas Terrados, Jaime E. Sampaio

**Affiliations:** ^1^Department of Physical Education and Sports, Faculty of Education and Sport, University of the Basque Country, UPV/EHU, Vitoria, Spain; ^2^Tottenham Football Club, London, United Kingdom; ^3^National Institute of Physical Education of Catalonia, University of Barcelona, Barcelona, Spain; ^4^Manchester City Football Club, 1st Team, Manchester, United Kingdom; ^5^Academy Department, Deportivo Alavés, Vitoria-Gasteiz, Spain; ^6^Department of Health Sciences, Faculty of Health Sciences, Universitat Oberta de Catalonia, Barcelona, Spain; ^7^UCAM Research Center for High Performance Sport, Catholic University of Murcia (UCAM), Murcia, Spain; ^8^Faculty of Sport Sciences, Catholic University of Murcia (UCAM), Murcia, Spain; ^9^FC Barcelona Sports Performance Department, Barcelona, Spain; ^10^Núcleo de Alto Rendimento Esportivo de São Paulo (NAR), São Paulo, Brazil; ^11^Department of Biochemistry Molecular Biology and Physiology, Faculty of Health Sciences, Campus de Soria, University of Valladolid, Soria, Spain; ^12^National Institute of Physical Education of Catalonia, University of Lleida, Lleida, Spain; ^13^Department of Sports, Faculty of Physical Activity and Sports Sciences (INEF), Universidad Politécnica de Madrid, Madrid, Spain; ^14^Departamento de Medicina Deportiva, Fundación Deportiva Municipal de Avilés (FDM), Aviles, Spain; ^15^Research Center in Sports Sciences, Health Sciences and Human Development, CIDESD, University of Trás-os-Montes and Alto Douro, UTAD, Vila Real, Portugal

**Keywords:** soccer, injury, recovery, fatigue, preseason

Preseason is a period of critical importance to develop high-level performance in soccer, which is supposed to be conducted with the aim of maximizing players' participations in team training sessions (Windt et al., 2017). In fact, the development of technical, phycological, physical and tactical performance is particularly important to be addressed during this period (Ostojic, 2004; Di Salvo et al., 2007; Fessi et al., 2016), when a substantial number of new players (and sometimes coaches) are being integrated into the team and have to adapt to a typically new and different training process.

This specific period can be defined as the moment in which the main objective is the acquisition of individual and collective adaptations that allow starting the competition adequately. Over the years, it has been possible to optimize its development in blocks ranging from 4 to 6 weeks (Loturco et al., 2019). During this particular training phase, the optimization methods seek individualized strategies and the ability to adapt to changing training goals and workloads (Carey et al., 2018). From a conditioning point of view, the preseason is characterized by a high volume of training and a gradually increasing intensity (Nikolaos et al., 2015), however, team strategical and tactical training should also be a major determinant in this period. Recent research has reported that performance in large-sided games (8 vs. 8 with goalkeepers) has changed as an effect of the preseason training, expressed by the lower total distance covered (from 165.5 ± 23.8 to 142.2 ± 18.4 m.min^−1^) and, interestingly, by the higher movement synchronization percentages (Folgado et al., 2018). These results confirm that optimizing players' conditioning as well as team strategical and tactical attuning during the preseason might be a key determinant of future performances.

It is usual for teams to aim for the highest training loads during the preseason (Jeong et al., 2011). Accordingly, the competition carried during preseason is generally used with the purpose of progressively reaching adequate performance levels that may be maintained over the entire season. In fact, the competition periods can be very long (around 40 weeks) and sometimes including World and European Cups on summer or preliminary rounds for European Cups or Intertoto challenge. Therefore, the highest level of performance, congested fixtures are substantially increasing and requiring adequate care. In general, the competition period is followed by the off-season, consisting ~2–5 weeks, and the cycle is repeated, with players returning to the clubs for a successive preseason (Woods et al., 2002).

In these scenarios, a very important topic, is that the reality of modern soccer is presenting more and more marketing-related tours with commercial interest, with the preseason summer tournaments becoming less and less frequent. For example, in 2019, Real Madrid reported a 10-days preseason calendar fixtures during their USA tour as follows: July 20th played against Bayern Munich in Houston; July 23th played against Arsenal in Maryland; July 26th played against Atletico de Madrid in New Jersey, and finally, July 26th played against Tottenham in Berlin, Germany. Another example, in 2019, Manchester City reported an 11-days preseason calendar in China/Japan with four matches: July 16th played against West Ham in Nanjing; July 19th played against Wolves in Xangai; July 24 played against Kitchee in Hong Kong and, July 27th played against Yokohama Marinos in Yokohama.

It is a fact that these games are set closer and closer to the beginning of the preseason period, which may cause an extra pressure on the player and coaching staff, forcing them to accelerate the conditioning process, thus skipping important steps in the training process and implementing higher workload values than the expected for the period (Rabelo et al., 2016), probably with unwanted consequences at different levels. Furthermore, due to the high-level of these summer tournaments, the “need to win” is becoming an additional stressing factor (Lee et al., 2001) It has also been reported that competing with high-level opponents increases time-motion demands at high intensities in tandem with intra-team movement synchronization tendencies (Folgado et al., 2014).

It has been recently revealed that training load during the pre-season (6 weeks) of a professional elite football team may not have an effect on team physical performance during the first five official matches of the season, since no relationship was found between training load and injury rate, inflammation and muscle damage markers (Coppalle et al., 2019). One of the most significant findings from an initial Football Association's epidemiological study was the disproportionately high number of training injuries during July, a month that corresponds to the preseason period (Woods et al., 2002). Nevertheless, an inappropriate pre-season training period can be associated with a higher incidence of players' injuries throughout the competitive season (Eliakim et al., 2018).

In particular, quadriceps injuries were more frequent during preseason, whereas adductor, hamstring, and calf injury rates increased during the competitive season (Hägglund et al., 2013). In this sense from the institutions are considering to be able to manage the total volume of minutes per player quantifying all competitions in a season. Therefore, an extra effort to understand how elite players are adapting (acutely and chronically) to these new scenarios of being exposed to preseasons with more and more competitive matches is required.

In this sense, it seems appropriate to discuss a global phenomenon within the sports community: are preseason friendly matches, friendly in reality? And, which are the implications for in-season injury incidence? Bearing in mind the aforementioned arguments, this current phenomenon in soccer implies that:

a) Understanding soccer players' match-related fatigue and recovery profiles likely helps with developing conditioning programs that increase team performance and reduce injuries and illnesses (Silva et al., 2018).
b) The process of understanding the training effects should be analysed overcoming the isolated inspection of conditioning variables, thus, requiring the control of strategic and tactical-related variables which relate to team and players' performance (Folgado et al., 2018).
c) The resignation of the transitory period supposes that, let's not take advantage of how as a “*window of opportunity”* for players to both “recover” and “rebuild” for the following season (Silva et al., 2016).
d) Although players are under less pressure to win compared to the end-of-season (Weinberg et al., 2006) in the recent years, preseason games are less friendly.

There are many possible reasons why players who attend more preseason training weeks may have a higher risk of subsequent injury (Lee et al., 2001). Nevertheless, recent studies suggest that players are at the greatest risk during the pre-competition period, and the low preseason cumulative workloads seem to be associated with increased in-season injury risks (Colby et al., 2017). In addition, large increments in weekly training load seem to increase injury risk (Harrison and Johnston, 2017). In this regard, an interesting study by Ekstrand in 2016, using a prospective design of 255 hamstring injuries in the “UEFA Elite club injury study” concluded that the majority of the intramuscular injuries affected muscle function (56% in grade 1 and 2 injuries), but no difference in lay-off time was found between the different types of injuries (Ekstrand et al., 2016).

In summary, an urgent SOS to the world of soccer is required. The preseason games are becoming less and less friendly. An evidence-based analysis suggests that more and more intense competitions during the consecutive seasons require to respect the primary objective of preseasons, which is to properly prepare and conditioning the players after a period of decreased activity. This is a crucial phase in which the competition stress should to be moved to the background and the physical conditioning and cognitive readiness should be the focus, to increase the chances of reaching the important moments of the season in the peak performance, and with all players available (or, at least, with the greatest number available). As the ability to provide multi-peak performances over the season does not seem possible (Issurin, 2010) and this is a real necessity in modern soccer, this issue should be further discussed.

## Author Contributions

JC-G: first idea. CL, DM-J, PA, TF, and NT: review. FC, AG-D, JM, IL, XP, and IR: design. JS: final approval. All authors contributed to the article and approved the submitted version.

## Conflict of Interest

The authors declare that this text was conducted in the absence of any conflict of interest. Our institution did not receive payment or services from a third party for any aspect of the submitted work and we declared no financial relationships with entities that could be perceived to influence, or that give the appearance of potentially influencing.
